# Influence of Pyrexia on Pharmacokinetics of Azithromycin and Its Interaction With Tolfenamic Acid in Goats

**DOI:** 10.3389/fvets.2021.675603

**Published:** 2021-06-10

**Authors:** Sini Mechery, Suresh Narayanan Nair, Thirumangalath Meethal Divya, Kanjirakuzhiyil Promod, Sakkariya Ibrahim Nalukudy Paramba, Reghu Ravindran, Sanis Juliet

**Affiliations:** ^1^Department of Veterinary Pharmacology and Toxicology, College of Veterinary and Animal Sciences, Pookode, Kerala Veterinary and Animal Sciences University, Wayanad, India; ^2^Department of Animal Reproduction Obstetrics and Gynaecology, College of Veterinary and Animal Sciences, Pookode, Kerala Veterinary and Animal Sciences University, Wayanad, India; ^3^Department of Livestock Production Management, College of Veterinary and Animal Sciences, Pookode, Kerala Veterinary and Animal Sciences University, Wayanad, India; ^4^Department of Veterinary Parasitology, College of Veterinary and Animal Sciences, Pookode, Kerala Veterinary and Animal Sciences University, Wayanad, India

**Keywords:** blood concentration, non-compartmental, half-life, HPLC, clearance, mean residence time

## Abstract

Azithromycin is a macrolide antimicrobial agent of the azalide group with a broad spectrum of activity against gram-negative and gram-positive bacterial organisms. Tolfenamic acid is a non-steroidal anti-inflammatory drug of the fenamate group, which is used extensively in humans and animals due to its anti-inflammatory, analgesic, and antipyretic properties. There is dearth of literature on any type of drug interaction between azithromycin and tolfenamic acid in any species, including human beings and alteration of its pharmacokinetics by fever. Therefore, the objective of this study was to investigate the alteration of disposition kinetics of azithromycin alone and in the presence of tolfenamic acid in Malabari goats by fever, following an intravenous administration at a dose rate of 20 mg/kg body weight. Blood samples collected from both afebrile and febrile goats at predetermined time intervals after the administration of azithromycin alone and then in combination with tolfenamic acid (2 mg/kg, intravenously), respectively, were analyzed using high-performance liquid chromatography. Non-compartmental analysis was used to determine the peak blood concentration (*C*_max_), time-to-peak plasma concentration (*T*_max_), half-life (*t*_1/2λ*z*_), area under the curve (AUC _0−t_, AUC _0−inf_), area under the first moment curve (AUMC _0−inf_), mean residence time (MRT_0−inf_), apparent volume of distribution at steady state (*V*_ss_), and the total body clearance of drug from the blood (Cl). In febrile animals, significant differences were noted in the values of *C*_max_, Cl, and *V*_ss_. Thus, azithromycin disappears into an additional compartment in febrile goats, which may be due to its extended cellular penetration into the inflammatory cells, resulting in anti-inflammatory activity. Tolfenamic acid significantly altered the pharmacokinetics of azithromycin in both normal and febrile animals. Tolfenamic acid, being a better anti-inflammatory agent, suppresses the inflammatory mediators, reducing the possibility of increased utilization of azithromycin in febrile condition.

## Introduction

Azithromycin is a macrolide antimicrobial agent of the azalide group which differs structurally from other macrolides by the insertion of a methyl-substituted nitrogen in its lactone ring. This structural modification significantly increased its potency against gram-negative bacteria when compared to erythromycin, with retention of activity against gram-positive bacteria (1). Azithromycin exerts its antimicrobial activity against a large number of gram-positive and gram-negative aerobic and anaerobic species that are associated with respiratory, skin, and sexually transmitted infections. Azithromycin is of major importance in the treatment of intracellular pathogens such as *Chlamydia pneumoniae, Chlamydia trachomatis, Mycoplasma pneumoniae*, and *Legionella pneumophila* (2) as well as enteric pathogens such as *Salmonella typhi, Shigella flexneri, Shigella sonnei*, and *Shigella dysenteriae* (3).

Tolfenamic acid is a non-steroidal anti-inflammatory drug (NSAID) of the fenamate group. It is used extensively in humans, dog, cat, calf, and pig medicine for its anti-inflammatory, analgesic, and antipyretic properties. Its anti-inflammatory and anti-endotoxemic properties are also used to enhance the rate of recovery in combination with antimicrobial drugs in acute mastitis in cattle, in pneumonia, and other viral and bacterial respiratory diseases in calves and in pigs for treatment of the metritis–mastitis–agalactia syndrome (4). Tolfenamic acid and azithromycin are licensed for the therapy of calf respiratory disease.

Goat is a minor species for which very few drugs are licensed for clinical use. However, as in calves, pneumonia in goat is a condition which requires therapy, and antimicrobial drugs are likely to be used both alone and in combination with NSAIDs. There is a dearth of literature on the pharmacokinetics and pharmacodynamics of drugs of both classes in goats. Moreover, from the limited published literature in this field, it is clear that data cannot be transposed from species such as the calf to the goat (5, 6).

The pharmacokinetics of azithromycin has been well-characterized in healthy volunteers, including humans (7), rats and dogs (8), cats (9), sheep and goats (10, 11), rabbits (12), and horses (13), with very few investigations in diseased subjects. The pharmacokinetics of different antimicrobials are reported to vary during different disease conditions (14, 15). Fever is the most important clinical manifestation of many infectious diseases in goats and is reported to induce biochemical and physiological alterations in cells (16, 17). The changes in the pharmacokinetic profile of many drugs during fever may influence the efficacy of antibacterial therapy (18, 19). In order to obtain the optimal efficacy of a drug, it becomes necessary to modify its dosage regimens on the basis of pharmacokinetic data of the drug obtained during the actual disease state (20). The effect of induced fever on the pharmacokinetics of other antimicrobials, *viz*., chloramphenicol (21), oxytetracycline (22), norfloxacin (23), and ceftriaxone (24), was previously described in goats.

Despite the great potential for its clinical use in veterinary medicine either alone or in combination with NSAIDs, the effect of fever on the pharmacokinetics of azithromycin after an intravenous administration in goats has not been studied so far. Therefore, the present study was aimed to determine the blood concentration and pharmacokinetic parameters of azithromycin alone and after co-administration with tolfenamic acid in Malabari breed of goats following its intravenous administration and its alteration in febrile condition.

## Materials and Methods

All the solvents, standards, and chemicals used in this study were of chromatographically pure grade and procured from M/s Merck India Ltd. and M/s Sigma-Aldrich India Ltd., Bangalore. The water used in all experiments was purified on a Milli-Q® system from M/s Millipore (Bedford, MA, USA).

The high-performance liquid chromatography system (HPLC; M/s Shimadzu Corporation, Kyoto, Japan), equipped with a LC-10AT quaternary gradient pump, a Rheodyne manual loop injector 20 μl, a column oven CTO-10AS VP, a diode array detector SPD- M20A, and a class-VP 6.12 version software for data analysis, was used for the detection and quantitation of azithromycin. To finalize the analytical protocol by reverse-phase HPLC, two procedures (25, 26) were considered in the present study. The mobile phase used for the detection of azithromycin was comprised of a mixture of potassium dihydrogen phosphate buffer, with the pH adjusted to 7.5 with 10% sodium hydroxide and methanol (20:80) at a flow rate of 1.7 ml/min and detector wavelength at 215 nm.

Ascending standards of azithromycin, that is, 100, 200, 300, 400, 500, 600, 800, 1,000, and 2,000 μg/ml in methanol, were determined using HPLC. A linear regression was performed on the data set, and a regression coefficient (*R*^2^) of 0.9988 was obtained for azithromycin.

Recovery from blood was achieved after fortifying the blood with different concentrations of azithromycin such as 50, 500, 1,000 and 5,000 μg/ml using methanol, and the precision and accuracy were calculated.

Twenty-four clinically healthy adult male and non-pregnant female goats (breed: Malabari, 1.5–2 years in age), weighing between 20 and 30 kg and procured from the Instructional Livestock Farm of College of Veterinary and Animal Sciences, Pookode, Wayanad, Kerala, India, were used in the present study, and the experiments were approved by the institutional animal ethics committee of the same college. The male and female goats were kept in different cages and provided with water *ad libitum*. The animals were given standard feed pellet in the morning and green grass, two times in a day, as recommended in the farm routine. During the initial observation period of 7 days, the body temperature, pulse, respiration, rumen motility, defecation, urination, and any other abnormality noted were recorded daily. Prior to the actual experiment, three goats were selected, and fever was induced by an intravenous injection of endotoxin (lipopolysaccharide or LPS from *E. coli*, serotype O127: B8, supplied by M/s Sigma-Aldrich India Ltd., Bangalore) at a dose rate of 0.2 μg/kg body weight. The temperature was recorded at different time intervals up to 12 h. At the fifth hour, half of the initial dose (0.1 μg/kg) was administered i.v. to each animal to prolong and maintain the pyrogenic effect for 12 h.

Twenty-four animals were randomly divided into four groups consisting of six animals each. The experimental design and the doses used are presented in Table 1. Azithromycin alone and azithromycin with tolfenamic acid were administered intravenously to normal healthy afebrile goats of group I and group II, respectively. To febrile goats of group III and group IV, azithromycin alone and azithromycin with tolfenamic acid, respectively, were administered intravenously at 1 h after the injection of endotoxin which was to induce the febrile condition.

**Table 1:** Experimental protocol.

**Treatment**	**Group**	**Drug**	**Dose**	**Route**
Afebrile (normal) group	I	Azithromycin	20 mg·kg^−1^	i.v
	II	Azithromycin	20 mg·kg^−1^	i.v
		Tolfenamic acid	2 mg·kg^−1^	
Febrile group	III	Azithromycin	20 mg·kg^−1^	i.v
		Lipopolysaccharide	0.2 μg·kg^−1^	
	IV	Azithromycin	20 mg·kg^−1^	i.v
		Tolfenamic acid	2 mg·kg^−1^	
		Lipopolysaccharide	0.2 μg·kg^−1^	

*i.v., intravenously*.

For the administration of azithromycin, the right jugular vein of each animal was cannulated by “Venflon 2” intravenous cannula with an injection port and flushed with heparin (0.04%) solution as and when required. Azithromycin (gifted by M/s Intas Pharmaceuticals Ltd., India) was injected intravenously at a dose rate of 20 mg/kg to each of the six normal goats of groups I and II. In addition, each animal of group II was also administered with tolfenamic acid intravenously at a dose rate of 2 mg/kg. Blood samples were collected into heparinized tubes by left jugular venepuncture of each goat at 0 (control), 5, 10, 15, 30, and 45 min and at 1, 1.5, 2, 4, 6, 8, 12, 24, 36, 48, 72, 96, 120, 144, and 168 h of drug administration and stored at −20°C until further processing and estimation by HPLC.

Fever was induced experimentally by an intravenous injection of the pyrogen (LPS) to each of the six goats of groups III and IV. Azithromycin was administered at febrile phase 1 h post-injection. Each animal of group IV was also administered tolfenamic acid intravenously at a dose rate of 2 mg/kg. Blood samples were collected at the same pre-determined time intervals as in groups I and II. In addition, the rectal temperature of each animal was recorded at 30-min intervals throughout the period of blood sample collection.

Whole blood samples were collected after de-proteinization with 2.5 ml of saturated ammonium sulfate, and azithromycin was extracted with methanol and reduced to a final volume of 1 ml for subsequent HPLC analysis.

The blood levels of azithromycin of each goat of groups I, II, III, and IV were plotted on a semi-logarithmic paper against time and analyzed using PKsolver software (27–29). The mean and standard error of each pharmacokinetic parameter was determined (30). One-way ANOVA with Tukey's multiple comparison as *post hoc* was done using GraphPad Prism (V 5.01).

## Results

**Figure 1:**
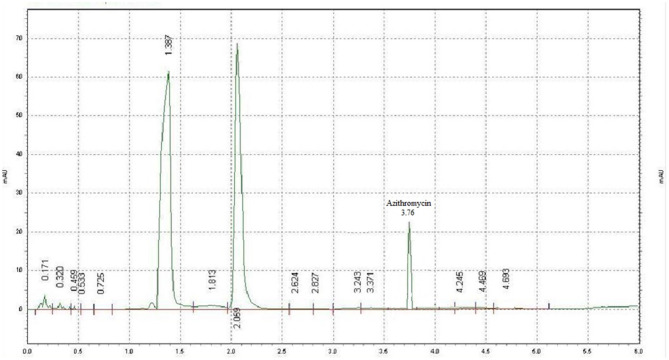
Chromatogram of azithromycin (analytical standard).

**Figure 2:**
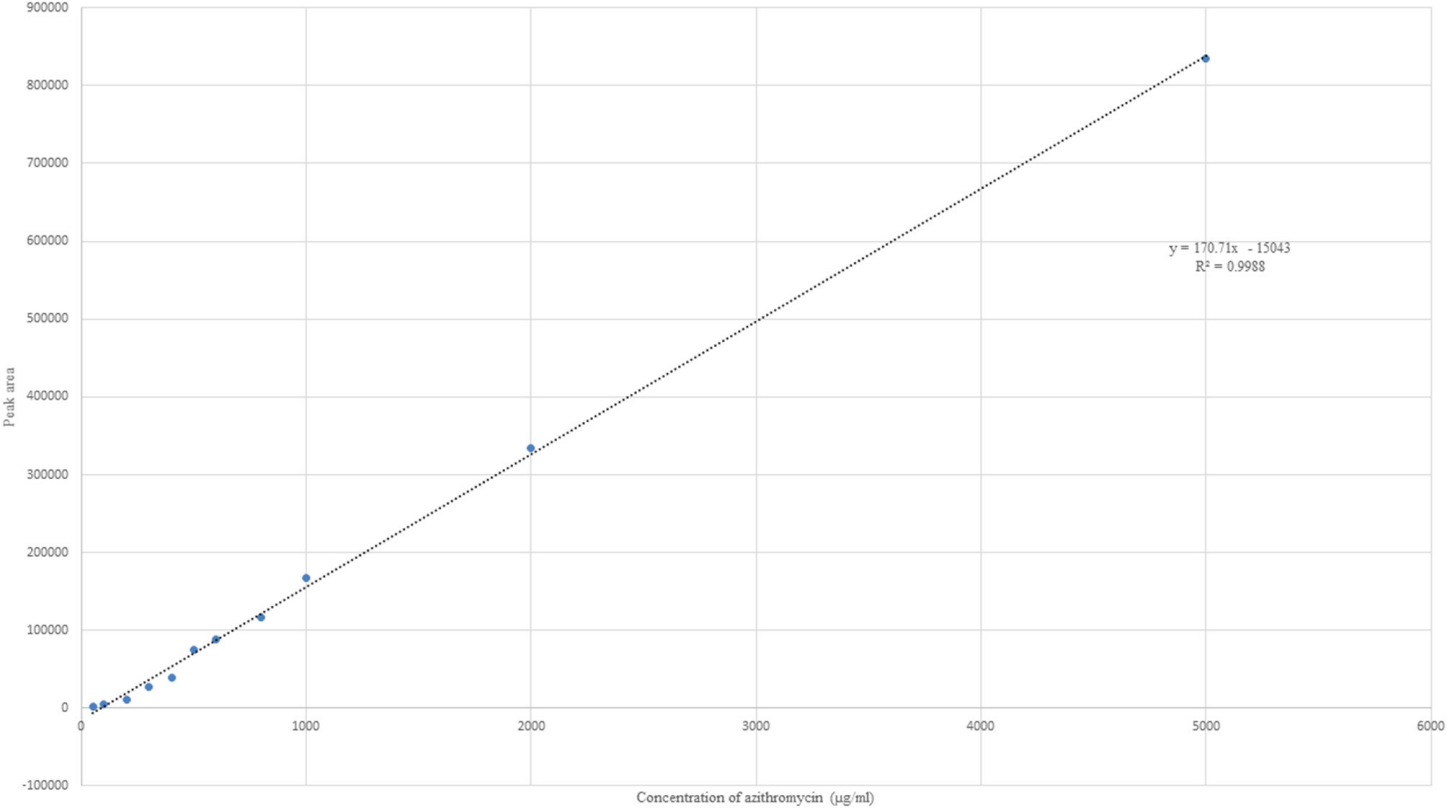
Linear plot of concentration vs. area for azithromycin with linear regression equation and *R*^2^ value.

Azithromycin was detected with potassium dihydrogen phosphate buffer and methanol (20:80) at a flow rate of 1.7 ml/min and detector wavelength of 215 nm. A sharp peak was detected at 3.76 min (Figure 1). Ascending standards of azithromycin (100, 200, 300, 400, 500, 600, 800, 1,000, and 2,000 μg/ml in methanol) were injected; the peaks with area were calculated, and a calibration curve of concentration vs. area was plotted (Figure 2). A linear regression was performed on the data set, and a regression coefficient (*R*^2^) of 0.9988 was obtained for azithromycin.

Recovery from blood after fortifying with 50, 500, 1,000, and 5,000 μg/ml concentrations of azithromycin was done. It was found that the extraction with methanol yielded a maximum recovery percentage ranging from 90.41 ± 4.14 to 98.12 ± 3.12% for concentrations ranging from 50 to 5,000 μg/ml (Table 2). The percent recovery of azithromycin ranged from 85 to 95%, and the relative standard deviation (RSD) was 0.4–4%, which were within the criteria of Codex Alimentarius Commission for residue analysis, that is, recovery of 70–110% and RSD of <20%.

**Table 2:** Recovery percentage of azithromycin and its relative standard deviation from fortified blood (*n* = 6, mean ± SE).

**Spiked concentration (μg/ml)**	**Recovery percentage**	**Relative standard deviation**
50	90.41 ± 4.14	10.24
500	95.22 ± 3.02	7.09
1,000	96.13 ± 2.19	5.09
5,000	98.12 ± 3.12	3.18

In the normal afebrile animals, rectal temperature varied within the normal range during the entire experimental period. In febrile animals, a rise in rectal temperature (102–104°F) was observed 1 h after LPS administration, and it remained elevated up to 12 h with the subsequent dose.

**Figure 3:**
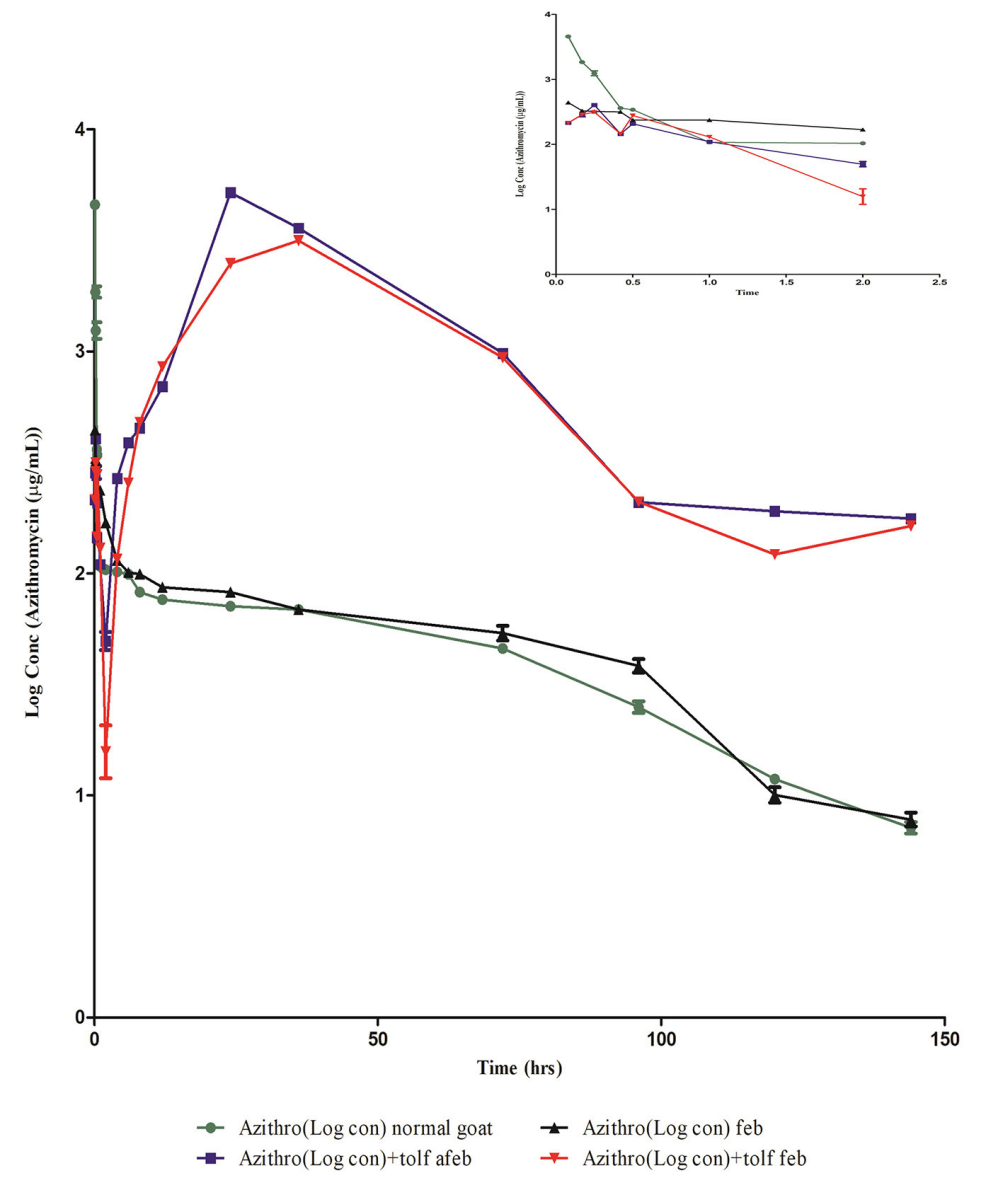
Semi-logarithmic plot of blood concentration (mean ± SE) vs. the time curve of azithromycin administered alone and in combination with tolfenamic acid intravenously to normal afebrile and febrile Malabari goats (*n* = 6).

The semi-logarithmic plot and mean (±SE) blood concentration of azithromycin after a single intravenous administration of azithromycin alone and in the presence of tolfenamic acid in normal and febrile animals is shown in Figure 3 and Table 3, respectively. The drug was present in detectable concentrations at all collection times between 5 min and 144 h in all the groups. In normal animals, maximum azithromycin concentrations were achieved at 0.08 and 24 h after the administration of azithromycin alone (group I) and in the presence of tolfenamic acid (group II), respectively, and the corresponding *C*_max_ values were 4,613.56 ± 211.474 and 5,211 ± 169.19 μg/ml (Table 3). In group I, the mean blood concentration of azithromycin at 5 min quickly reduced to one-third of its value in the next 5 min (1,868.105 ± 103.405 μg/ml), which continued to descend till 30 min, followed by a steady decline. On the other hand, the initial blood concentrations of azithromycin in the presence of tolfenamic acid (group II) were much lesser than when azithromycin was administered alone. The blood concentration, after an initial ascent, steadily decreased from 15 min to 2 h, followed by a gradual increase in the concentration up to 24 h. At 24 h, the concentration reached *C*_max_ (5,211 μg ml^−1^), which was almost 20 times the concentration of azithromycin at 5 min. This was further followed by a steady decline in concentration up to 144 h. From 4 h onwards, the concentrations were significantly higher in goats receiving azithromycin in the presence of tolfenamic acid. The concentration of azithromycin in the presence of tolfenamic acid at 144 h remained significantly high than when administered alone.

**Table 3:** Mean (±SE) blood concentration (μg/ml) of azithromycin after single intravenous administration of azithromycin alone and in the presence of tolfenamic acid in normal and febrile goats.

**Time (h)**	**Blood concentration (μg/ml) of azithromycin (n** **=** **6, mean** **±** **SE)**			
	**Normal**		**Febrile**	
	**Group I**	**Group II**	**Group III**	**Group IV**
0.08	4, 613.56 ± 211.47	215.74 ± 7.35	441.79 ± 5.05	215.74 ± 5.72
0.17	1, 868.11 ± 103.41	288.54 ± 20.58	330.66 ± 7.89	288.82 ± 5.51
0.25	1, 267.42 ± 121.43	404.5 ± 7.06	321.79 ± 5.91	315.11 ± 5.78
0.42	363.96 ± 16.99	146.15 ± 7.37	317 ± 5.45	146.44 ± 6.31
0.5	343.73 ± 15	208.00 ± 3.76	238.66 ± 8.49	278.78 ± 5.54
1	108.00 ± 3.57	110.01 ± 2.57	237.68 ± 6.34	130.83 ± 5.6
2	104.72 ± 4.96	50.71 ± 4.43	169.33 ± 3.75	19.06 ± 5.5
4	102.00 ± 3.69	269.01 ± 7.07	115.5 ± 5.76	116.96 ± 6.24
6	99.82 ± 4.41	390.00 ± 13.78	101.37 ± 3.87	255.81 ± 5.74
8	83.00 ± 4.16	453.02 ± 12.32	99.93 ± 4.49	480.14 ± 7.08
12	76.35 ± 2.04	694.50 ± 7.44	87.26 ± 4.22	855.00 ± 5.79
24	71.34 ± 2.03	5.211 ± 169.19	82.87 ± 3.69	2, 489.6±14.07
36	68.95 ± 1.89	3, 597.02 ± 65.61	69.2 ± 3.35	3, 155.87±29.57
48	55.34 ± 1.13	983.01 ± 13.67	54.61 ± 3.92	938.83 ± 5.41
72	46.08 ± 1.41	210.01 ± 7.21	38.84 ± 2.67	211.1 ± 7.82
96	25.25 ± 1.5	191 ± 5.04	10.21 ± 0.81	122.45 ± 6.14
120	11.89 ± 0.48	178.03 ± 8.56	7.89 ± 0.55	164.00 ± 5.52
144	7.22 ± 0.45	48.01 ± 3.66	6.76 ± 0.45	59.2 ± 5.41

However, in febrile animals, the maximum mean blood azithromycin concentrations were achieved at 0.08 and 36 h after administration of azithromycin alone and in the presence of tolfenamic acid, respectively, and the corresponding *C*_max_ values were 441.79 ± 211.474 and 3,155.87± 29.57 μg/ml (Table 3). In febrile animals administered with azithromycin alone, the mean blood concentration of azithromycin at 5 min progressively reduced, and the lowest concentration was detected at 114 h. Though the *C*_max_ was low compared to that of normal goats of group I, fever did not significantly alter the azithromycin concentrations from 6 h onwards. On the other hand, the initial blood concentration of azithromycin in the presence of tolfenamic acid in the febrile group elicited an initial ascent, followed by a steady decline from 15 min to 2 h, followed by a gradual increase in concentration up to 36 h. At 36 h, the concentration reached *C*_max_ (3,155.87 ± 29.57 μg ml^−1^), which was almost 15 times the concentration of azithromycin at 5 min. This was further followed by a sharp decline in concentration up to 144 h. However, in febrile animals, the blood concentrations were significantly higher in goats receiving azithromycin in the presence of tolfenamic acid from 6 h onwards, and at 144 h, the concentration remained on the higher side.

A non-compartmental analysis was done for deriving the pharmacokinetic parameters in all the animals of treatment groups I, II, III, and IV. The mean non-compartmental pharmacokinetic parameters of azithromycin in normal and febrile Malabari goats after a single intravenous administration of azithromycin alone and in the presence of tolfenamic acid are summarized in Table 4.

**Table 4:** Non-compartmental pharmacokinetic parameters (mean ±SEM) in various treatment groups of azithromycin administration in Malabari goats.

**Parameter**	**Normal afebrile**		**Febrile**	
	**Group I**	**Group II**	**Group III**	**Group IV**
	**Azithromycin alone**	**Azithromycin tolfenamic acid**	**Azithromycin alone**	**Azithromycin with tolfenamic acid**
λ_z_ (1/h)	0.026 ± 0.003^a^	0.037 ± 0.0005^b^	0.02 ± 0.001^a^	0.032 ± 0.0007^b^
t_1/2__λz_ (h)	28.35 ± 3.067^a^	18.96 ± 0.238^ab^	35.91 ± 3.471^a^	21.66 ± 0.463^b^
T_max_ (h)	0.08 ± 0	24 ± 0	0.08 ± 0	36 ± 0
C_max_ (μg/ml)	4, 613.56 ± 211.474^a^	5, 211±169.19^c^	441.79 ± 4.12^b^	3, 155.87±29.57^d^
C_last__/C_max_	0.002 ± 0^a^	0.009 ± 0.0007^c^	0.015 ± 0.001^b^	0.019 ± 0.0017^b^
AUC_0−t_ (μg h/ml)	7, 119.62 ± 47.48^a^	146, 427.17±2, 435.17^b^	6, 334.08±222.45^a^	106, 630.28±1, 101.15^c^
AUC_0−inf_ (μg h/ml)	7, 424.74 ± 70.04^a^	147, 744.95±2, 481.75^b^	6, 682.68±242.76^a^	108, 498.55±1267.97^c^
AUC_0−t_/AUC_0−inf__	0.959 ± 0.007^a^	0.991 ± 0.0007^b^	0.948 ± 0.004^a^	0.983 ± 0.0018^b^
AUMC_0−inf__ (μg h/ml)	352, 152.00 ± 12, 807.95^a^	5, 613, 272.74±90, 915.60^b^	308, 997.96±16, 525.924^a^	4, 538, 985.30±102, 832.07^c^
MRT_0−inf__ (h)	47.39 ± 1.38^a^	38.002 ± 0.33^b^	46.06 ± 1.09^a^	41.81 ± 0.49^b^
V_ss_ (L/kg)	1.10 ± 0.11^a^	0.037 ± 0.0008^c^	1.55 ± 0.13^b^	0.058 ± 0.0008^c^
Cl (ml/kg/h)	0.027 ± 0^a^	0.001 ± 0^c^	0.03 ± 0.001^b^	0.002 ± 0^c^

*The pharmacokinetic parameters are calculated with PKSolver MS_Excel add-in. One-way ANOVA with Tukey's multiple comparison as post hoc was done using GraphPad Prism (V 5.01). Values with different superscripts (a, b, c, d) vary significantly across the rows (n = 6).*

*λ_z_, terminal slope of a semilogarithmic concentration–time curve; t_1/2λz_, terminal half-life; T_max_, time of maximum blood concentration; C_max_, maximum blood concentration; C_last_/C_max_–ratio of the last blood concentration and maximum blood concentration; AUC_0−t_, area under the curve from zero to time t; AUC_0−inf_, area under the curve from zero to infinity; AUMC_0−inf_, area under the first moment curve; MRT_0−inf_, mean residence time; V_ss_, apparent volume of distribution at steady state; Cl, total body clearance of drug from the blood*.

The non-compartmental pharmacokinetic parameters of azithromycin, when compared between normal and febrile goats after a single intravenous administration of azithromycin, however, did not show much significant differences in *t*_1/2__λ*z*_, AUC 0-t, and MRT values. Though the peak concentration *C*_max_ was not comparable (4,696.19 ± 248.097 and 441.79 ± 4.122 μg/ml), the corresponding time (*T*_max_) was 0.08 h in normal and febrile animals, respectively. The *t*_1/2__λ*z*_ and MRT values were 28.35 ± 3.067 and 47.39 ± 1.38 h, respectively, for normal animals and 35.91 ± 3.471 and 46.06 ± 1.09 h, respectively, for febrile animals. However, a significant difference was noted for the observed clearance and steady-state volume of distribution. The observed clearance was 0.027 ± 0 and 0.03 ± 0.001 ml/kg/h, and the steady-state volume of distribution was 1.10 ± 0.11 and 1.55 ± 0.13 ml/g in normal and febrile animals, respectively.

The pharmacokinetic analysis of azithromycin co-administered with tolfenamic acid in both normal and febrile groups revealed that tolfenamic acid altered the kinetics of azithromycin. Literature search, however, did not provide any evidence on drug interaction between azithromycin or other macrolides and tolfenamic acid in any species including human beings. In the presence of tolfenamic acid, azithromycin attained a *C*_max_ value of 5,211 ± 169.19 μg/ml at 24 h in group II animals when compared to *C*_max_ of 4,613.56 ± 211.47 μg/ml achieved at 0.08 h in group I. Besides this, there was also a significant difference in the observed values of steady-state volume of distribution (Vss_obs_) and clearance (Cl_obs_) between groups I and II. Moreover, *t*_1/2__λ*z*_ was 18.96 ± 0.238 h in group II compared to 28.35 ± 3.067 h in group I. Similarly, there was a significant difference in the *C*_max_ value and corresponding *T*_max_ values, observed values of AUC_0−*t*_, MRT, steady-state volume of distribution, and clearance between groups III and IV. Furthermore, the non-compartmental pharmacokinetic parameters of azithromycin, when co-administered with tolfenamic acid in normal and febrile goats, did not show much significant difference except for the peak concentration (*C*_max_) and the corresponding *T*_max_, AUMC _0−inf_, and AUC_0−*t*_ values.

## Discussion

Fever is the most important clinical manifestation of many infectious diseases in goats and is reported to induce biochemical and physiological alterations in cells (16, 17). In the present study, LPS injection in goats (0.2 μg/kg intravenously) produced fever (102–104°F) for 5 h and was maintained for 12 h by giving a further half dose (0.1 μg/kg intravenously) at the fifth hour. It is established that when lipopolysaccharide is injected by the intravenous route, it interacts with various cellular components such as neutrophils, basophils, eosinophils, monocytes, and other fixed macrophages and releases the endogenous pyrogen responsible for producing a febrile condition (31). Up to 1 mg of LPS has been given to sheep to evaluate changes in pharmacokinetic values (18), whereas 0.1 μg/kg has been given to horses, rabbits, and dogs (18, 32).

Azithromycin was already reported (33) to be taken up by the formed elements of the blood (white cells, red cells, and platelets), and therefore the plasma concentration of azithromycin may be much less or negligible than the whole blood concentration. Fibroblast is the known reservoir of azithromycin, and the serum concentration could also be very low, so we measured the drug concentration in the whole blood.

The pharmacokinetics of azithromycin after intravenous administration alone and in combination with tolfenamic acid in normal and febrile goats was described by non-compartmental analysis. Intravenously administered azithromycin alone has been reported to fit a three-compartment model in goat (11), sheep (10), and rabbit (12) and to a non-compartmental model following intramuscular administration in rabbit and cow (34). In the present study, fever altered the distribution and total body clearance of azithromycin, even though not much significant change was noticed with other pharmacokinetic parameters, *viz*., *T*_max_, *t*_1/2λ*z*_, AUCs, AUMC, and MRT.

Correlating with the above-mentioned findings, two reasons may possibly be proposed for the altered pharmacokinetics of azithromycin in fever: first, an increased accumulation of azithromycin in macrophages by phagocytosis in febrile condition, and second, an increase in the number of neutrophils and monocytes that can accumulate azithromycin in febrile state. When LPS is injected, there will be an increased release of membrane phospholipids like phosphatidylcholine inside the cells, which are the raw materials for the synthesis of all the mediators of inflammation. Azithromycin, by virtue of its anti-inflammatory property, inhibits the lysosomal phospholipase A and binds with the released phospholipids (33, 35–38). These processes are comparatively stable, and azithromycin, once bound, may not diffuse back into plasma. Furthermore, in febrile state, there is a surge in the number of inflammatory cells (neutrophils and monocytes) as the production of granulocyte macrophage-colony stimulating factor is increased by LPS which, in turn, takes up larger quantities of azithromycin (39). These factors might be attributed to the reduction in blood concentration of azithromycin observed immediately after the injection in febrile state. On the other hand, in non-febrile conditions, the accumulated azithromycin is in a dynamic state, which is pumped back into the plasma by an efflux mechanism (40). Besides this, azithromycin, once internalized into the cell, undergo protonation and get entrapped in the acidic cytoplasm generated by the degradation of membrane phospholipid, which generate arachidonic acid that make the cytoplasm more acidic. This ion trapping, which occurs at a faster pace during an inflammation, may be the key factor that is responsible for a drastic fall in the initial concentrations of azithromycin in pyretic animals. This is further supported by a significant reduction of the *C*_max_ of azithromycin in febrile animals. Tolfenamic acid, on the other hand, can favor acidosis since the pKa value is acidic (3.88).

Azithromycin undergoes N-demethylation and O-demethylation and is excreted through the bile (2). Moreover, it is established that azithromycin may not interact with any cytochrome P450, unlike erythromycin. Hence, there may not be any probable reason related to its biotransformation, which may affect the pharmacokinetics either in normal or febrile animals.

One of the interesting observations from the present study is that, although tolfenamic acid can somehow alter the kinetics of azithromycin, it also protects from an alteration in the kinetics of azithromycin when given in pyrexia in contrast to what was observed in azithromycin-alone groups (groups I and III). Moreover, it is observed that there is a second peak plasma concentration of azithromycin (which actually becomes the *C*_max_ of azithromycin) when tolfenamic acid is co-administered with azithromycin. Correspondingly, there is a shift in the *T*_max_ to 36 h for azithromycin when it is co-administered with tolfenamic acid in both febrile and afebrile animals. Further studies are required for the exact reason for this observation.

By non-compartmental analysis, in febrile animals, the *C*_max_ of azithromycin was found to be reduced to 10.44% of the *C*_max_ of a normal animal. This is probably due to the fact that LPS can induce a greater number of circulating PMN cells, which will take up azithromycin from the blood. It can be inferred that, even though the inflammatory mediators increase the uptake of azithromycin by PMNL and monocytes, tolfenamic acid, being a better anti-inflammatory agent, suppresses the inflammatory mediators and then reduces the possibility of increased utilization of azithromycin when LPS is given. This may be the reason for the near-normal *C*_max_ in the case of tolfenamic acid co-administered group of animals. Azithromycin disappears from the plasma at a faster rate in the presence of tolfenamic acid, even faster than a pyretic animal, probably because tolfenamic acid favored the ion trapping of azithromycin even in the absence of inflammation (group II). This effect is seen again with MRT, which is least in tolfenamic acid group. Though the distribution of azithromycin is faster, its clearance is slower in the presence of tolfenamic acid both in febrile and afebrile animals. This paradoxical observation may be due to a shift in the dynamic equilibrium of the internalization and expulsion of azithromycin in the presence of tolfenamic acid.

The present investigation was undertaken to assess the changes in the pharmacokinetics of azithromycin in the presence of fever and tolfenamic acid. The study did not determine the pharmacokinetic parameters of tolfenamic acid after co-administration with azithromycin in order to establish any potential influence of azithromycin on the pharmacokinetics of tolfenamic acid. This is the major limitation of the current study being unable to analyze the pharmacokinetic interaction of tolfenamic acid in the presence of azithromycin.

From the above-mentioned findings, it can be concluded that fever, induced by a parenteral injection of exogenous pyrogen, has altered the distribution of azithromycin. The reduced blood concentration in pyrexia may be due to the extended cellular penetration of azithromycin into the inflammatory cells, which is also responsible for its anti-inflammatory activity. The co-administration of tolfenamic acid also significantly altered the disposition kinetics of azithromycin in normal and febrile goats.

## Data Availability Statement

The original contributions presented in the study are included in the article/supplementary material, further inquiries can be directed to the corresponding author/s.

## Ethics Statement

The animal study was reviewed and approved by the Institutional Animal Ethics Committee (IAEC), College of Veterinary and Animal Sciences, Pookode, Wayanad, Kerala, India.

## Author Contributions

SM and TD collected the samples, conducted the experiments, participated in the data acquisition, and drafted the manuscript. KP and SP helped in the collection of samples and data acquisition. SJ conceived the study, analyzed the kinetic parameters, and supervised the protocols. SN and RR helped in the collection of samples, data acquisition, calculations, supervision of the experiments, and review of the manuscript. All authors contributed to the article and approved the submitted version.

## Conflict of Interest

The authors declare that the research was conducted in the absence of any commercial or financial relationships that could be construed as a potential conflict of interest.
